# A Comprehensive Review of Nanogel-Based Drug Delivery Systems

**DOI:** 10.7759/cureus.68633

**Published:** 2024-09-04

**Authors:** Ferozekhan S, Marakanam S Umashankar, Damodharan Narayanasamy

**Affiliations:** 1 Department of Pharmaceutics, SRM College of Pharmacy, Faculty of Medicine and Health Science, SRM Institute of Science and Technology, Chengalpattu, IND

**Keywords:** swelling behavior, polymer, exceptional drug loading capability, drug delivery, nanogel

## Abstract

Polymers can be crosslinked chemically or physically to create three-dimensional hydrogel particles with sub-micron dimensions, known as nanogels. Their customizable size, ease of manufacture, expansion potential, bio-integration, water affinity, and reactivity to various stimuli, including temperature, pH, light, and biological agents, provide them with considerable advantages over conventional drug delivery techniques. Nanogels possess properties of both hydrogels and nanoparticles and can be categorized into nanohydrogels and nano-organogels. These systems exhibit exceptional drug-loading capability, stability, biological consistency, and environmental responsiveness. Their hallmark lies in their swelling behavior, enabling substantial water absorption while maintaining structural integrity. Preparation methods involve polymer precursors or heterogeneous polymerization of monomers. Nanogels are promising for various drug administration techniques, including local anesthetics, vaccines, and transdermal drug delivery, due to their ability to encapsulate multiple bioactive ingredients, enhancing therapeutic efficacy and stability.

## Introduction and background

Nanogel administration systems emerge as 3D hydrogel beads with a nanoscale dimension, formed through physical or chemical crosslinking of polymers. The internal polymer linking of polymers endows nanogels with the ability to absorb fluids while maintaining structural robustness without dissolving. Positioned as the future of drug delivery systems, nanogels offer distinct advantages over conventional systems, including tunable size, facile synthesis, expansion potential, physiological compatibility, hydration affinity, and responsiveness to environmental changes (temperature, pH, light, and biological agents). Although typically described as crosslinked polymer chains with sizes up to 100 nm, the accepted dimensions of nanogels may extend beyond 200 nm, frequently ranging from 1 to 1000 nm. When the polymers poly(ethylene glycol) and poly(ethyleneimine) were chemically crosslinked to create hydrophilic polymer networks for antisense oligonucleotide delivery, the name "NanoGel" was first used [1].

Nanogel possesses attributes of hydrogels and nanoparticles and can designated as polymer nanoparticles. They can be further classified into various types, including nanohydrogels and nano-organogels. Nanohydrogels, composed of hydrogels at the nanoscale, offer advantages such as biocompatibility, controlled drug release, and the ability to carry a variety of drugs. Nano-organogels, on the other hand, can hold oily substances and form micelle-like nanoparticulate systems. Nanogels exhibit exceptional drug-loading capability, stability, biological consistency, and responsiveness to environmental stimuli, distinguishing them from conventional pharmaceutical nanocarriers. The hallmark of nanogels lies in their swelling behavior, facilitated by polymer absorption of water, rendering them mostly hydrophilic and capable of incorporating substantial amounts of water or biological fluids while maintaining structural integrity. This swelling phenomenon occurs through interactions between the nano scaffold and solvent molecules, where polymer chains elongate upon water absorption until the elastic retroactive force balances the deformation, resulting in an equilibrium conducive to absorbing over 90% w/w of aqueous solution. Such water retention properties enhance the diffusion and exchange of ions, metabolites, and biomolecules, fostering synergy with biological applications and rendering nanogels promising biocompatible candidates.

Preparation approaches for nanogels involve formulating them from polymer precursors or synthesizing them through mixed polymerization of monomers. Polymers such as dual-surfactants serve as precursors for self-assembly, while polymers with active sites can be flexibly employed for chemical cross-linking. Nanogels offer the unique capability to encapsulate multiple bioactive ingredients in the same carrier, making them ideal for various drug delivery applications. In drug delivery, nanogels not only shield payloads from degradation and premature release but actively participate in the delivery process, owing to the judicious selection of polymers and architectural versatility allowing the incorporation of diverse molecules while maintaining gel-like behavior. The size of nanogels ranges from 20-200 nm. Because of their size, they can evade renal clearance and extend serum clearance half-life [2].

In summary, nanogels represent a versatile and promising approach to drug delivery, offering unique advantages such as synthesis, characterization, therapeutic applications, and prospects of nanogel-loaded drugs, elucidating their potential to revolutionize the field of drug delivery.

## Review

Classification of nanogels

**Figure 1:**
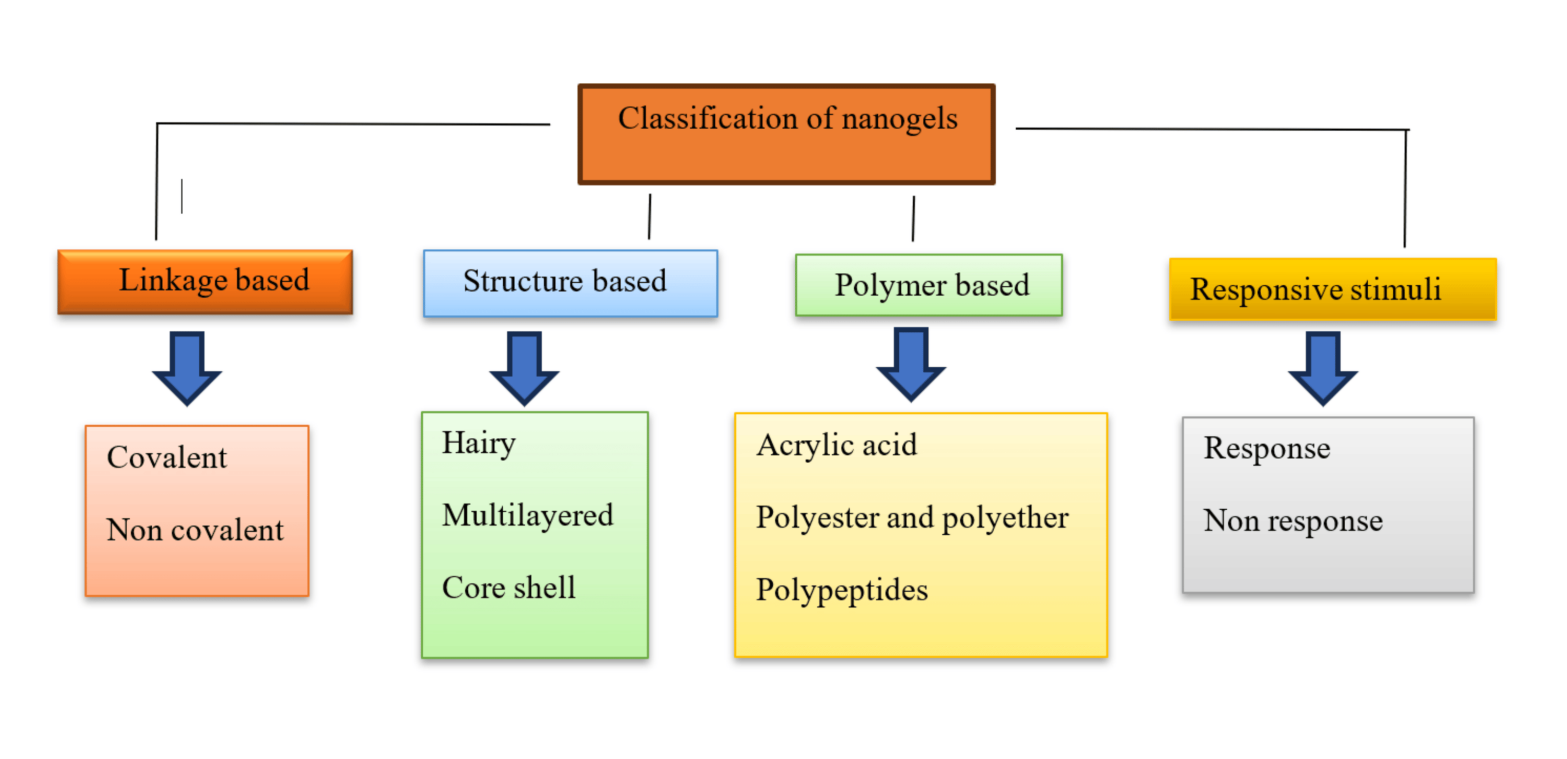
Classification of nanogels This image is an original creation by author Ferozekhan S.

The classification of nanogels is mentioned in Figure 1.

Based on Structure

**Figure 2:**
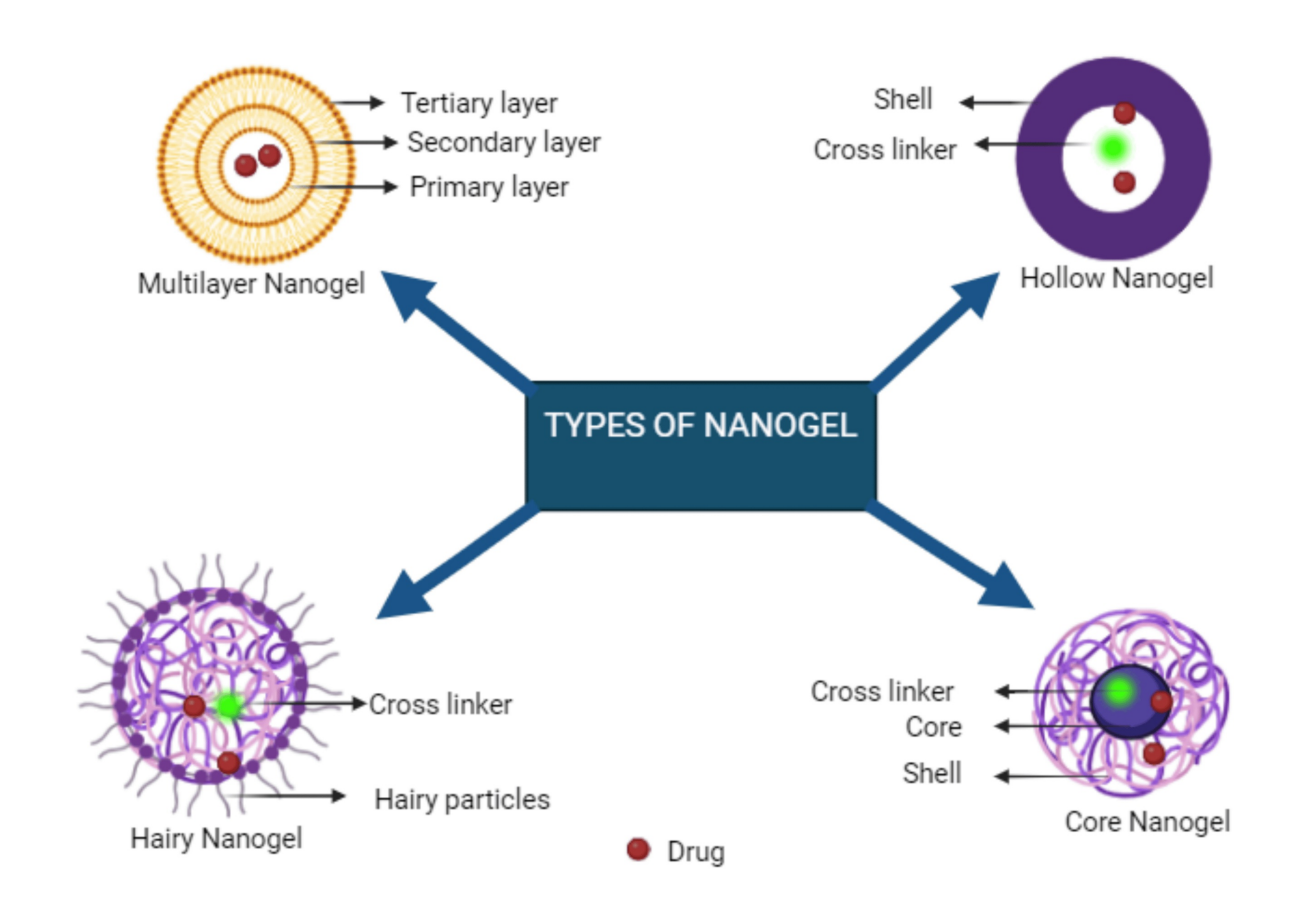
Types of nanogels based on structure This image is an original creation by author Ferozekhan S.

Nanogels can be classified according to their structural characteristics. Although typically spherical, nanogels can be synthesized. The types of nanogel based on structure are mentioned in Figure 2.

Hollow nanogels: These nanogels are prepared in two stages: first, polymers are joined together with core-shell particles (such as hydroxyl propyl cellulose, silicon dioxide, or gold), and then the core-shell moieties are extracted using pH gradients or anti-solvent precipitation methods. Within the gel of these nanogels is a hollow chamber. Precise removal is required for the intended hole to form. This increases surface area and makes hollow nanogels suitable for catalysis, medicine delivery, and water filtration.

Multilayered nanogels: These nanogels are made up of several layers of different polymers or one polymer. Obtaining high site specificity requires careful consideration of the polymer choices. Because the polymer arrangement influences the medication's release profile, this method works well for delivering extremely toxic or sensitive medications. Because multi-layered nanogels can be tuned to achieve high site specificity, they can be very helpful for addressing strong or low therapeutic effectiveness. However, their complexity, yield, scalability, and cost pose challenges for clinical application. They can also be used to deliver peptides, oligopeptides, and nucleotides. A novel gellan pullulan nanogel was synthesized using chemical crosslinking to remove methylene blue, benefiting from its multi-layer arrangement.

Core-shell nanogels: These systems consist of an exterior shell that encloses the inner core, which is usually composed of carbon dots, nanorods, or metallic or bimetallic materials. The outer portion can be polymeric chains or organic structures and can be positioned via chemical bonding or physical entrapment. This versatility allows tuning the system for various applications, such as drug delivery for proper payload release or sensors for specific responsive behavior [2].

Hairy nanogels: These angel surfaces are covered in fine, hair-like projections that are the result of either macroRAFT (reversible addition-fragmentation) agents or controlled radical polymerization. These projections are created by polymers that are covalently linked to the core gel matrix [2].

Based on Stimuli

Nanogels can be classified based on their responsiveness to various environmental stimuli.

Non-responsive nanogels: Non-responsive nanogels swell through simple absorption upon coming into contact with aqueous fluids, hence eliminating the need for external triggers to release drugs constantly at the target location [3].

Responsive nanogels: When environmental factors like electricity, pH, light, the magnetic field, ultrasound, strength of ions, or solvent composition change, responsive nanogels may swell or de-swell. These stimuli-responsive nanogels are frequently made from both natural and synthetic polymers, and they have a great ability to absorb water and swell. Response nanogels can quickly and reversibly go from a swelling to a collapsed state in response to various physical, chemical, or biological stimuli. Multi-responsive nanogels are defined as nanogels that respond to several stimuli. Responsive nanogels are a valuable substrate for biomedical applications because of their capacity to undergo reversal expansion and deswelling in response to diverse triggers. This feature enables regulated and targeted drug delivery [3].

Polymer-Based Classification

**Figure 3:**
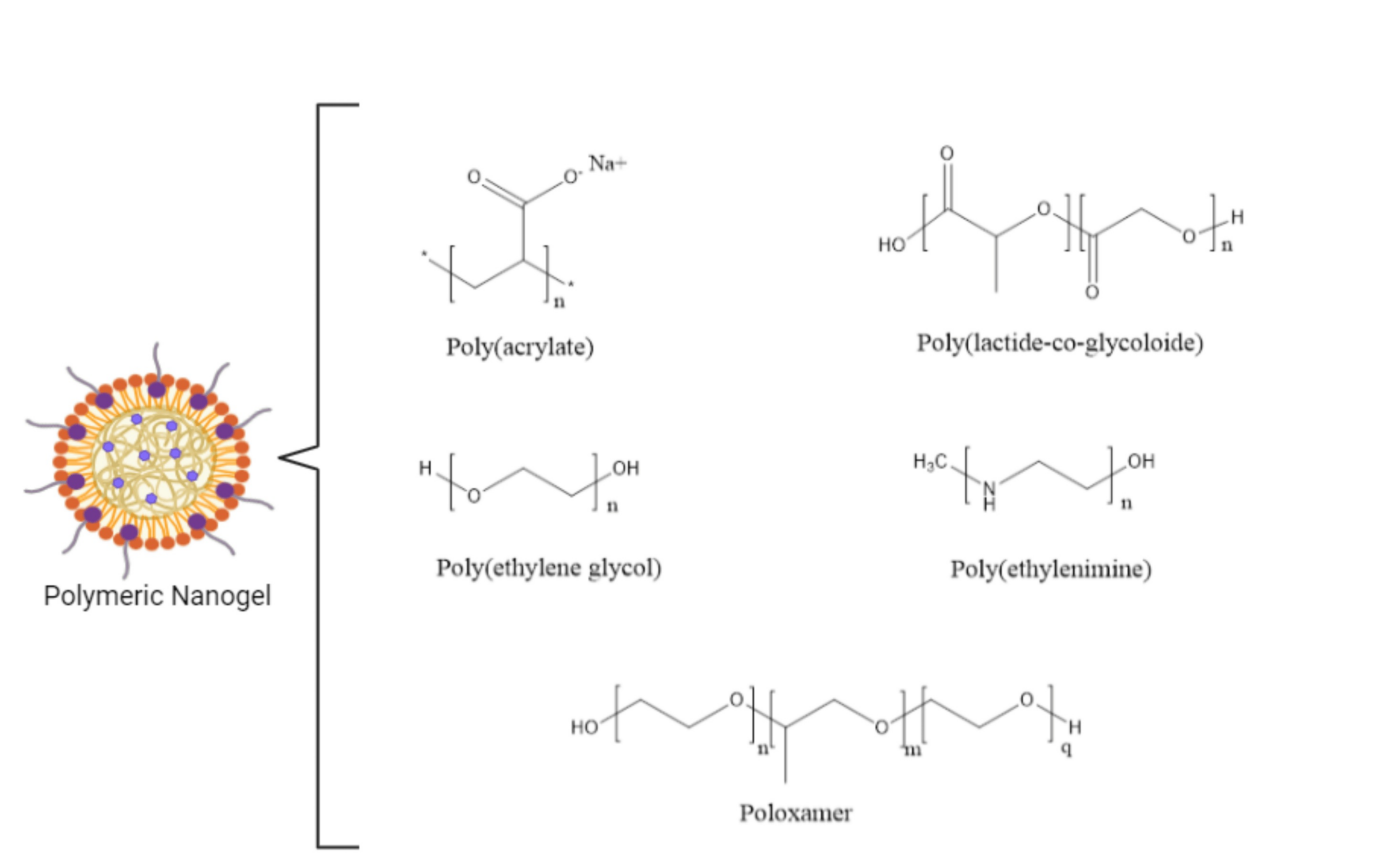
Types of nanogels based on polymers This image is an original creation by author Ferozekhan S.

Based on the kind of polymers used in their manufacture, nanogels can be categorized accordingly. The types of nanogels based on polymers are mentioned in Figure 3.

Polyacrylates: Acrylic acid derivatives are created by changing the vinyl and/or carboxyl chains. They display swelling behavior as a result of the external environment's counter-ion exchange.

Poly (ethylene oxide) and poly (ethylene terephthalate) (PET): Ethanol (R-O-R) and ester (RCOOR) connections, respectively, generate biodegradable polymers. Examples include polyglycolic acid, polylactic acid PLA, and their copolymer PLGA. PLA exists in D- and L-forms, with the L-form being more biodegradable.

Polyethylene glycol (PEG): A typical polyether polymer is highly biocompatible, water-soluble, stable, and biologically inert. It increases the circulation time by imparting hydrophilicity and reducing clearance

Poloxamers: It blocks the copolymers of polyethene oxide and polypropylene oxide. It is obtainable in different grades according to the polymer blocks' lengths.

Polypeptides: Amide bonds bind biodegradable polymers together. It has an ability to manipulate amino acid sequences during synthesis. Challenges include instability to hydrolysis and enzyme-dependent degradation.

Polysaccharides: Glycol polymers are between monosaccharide units. They can be homopolymers (e.g., starch, cellulose) or heteropolymers (e.g., heparin, chitosan). They possess multiple reactive groups for chemical and biochemical derivatization. They are water-soluble, non-toxic, biocompatible, biodegradable, and bioactive [4]. There are two primary categories for nanogels. Their responsiveness, which might be either stimulus-responsive or non-responsive, is the basis for the first classification. Water causes non-responsive nanogels to swell. Conversely, stimuli-responsive nanogels exhibit morphological changes in response to alterations in several environmental parameters, including but not limited to temperature, pH, magnetic fields, and ionic strength. Nanogels that are multi-responsive respond to multiple environmental stimuli. Polymeric gels, including nanogels, are classified into two primary types based on the type of connections present in the interconnected chains of the gel structure [4].

Classification Based on Linkage

Non-covalent linkage refers to self-assembling physical interactions that do not require cross-linking agents. Underlying mechanisms underlie surface/interface interactions, ionic interactions, adsorption, and Van der Waals forces, among others. Among its many benefits are its reversibility, lack of chemical interactions, and potentially hazardous bioactive substances or cells. However, weak connections are impeding their stability. Rigid covalent bond formation from exact stoichiometric processes is required for covalent linkage to create stable and repeatable couplings. It makes it possible to illuminate the components with the ability to alter while responding to their surroundings, allowing for controlled release [4].

Physically cross-linked nanogels: A combination of nanogel particles spread throughout an organic or inorganic matrix is referred to as a hybrid nanogel. Research has shown that polymer amphiphiles, such as pullulan-poly(N-isopropyl acrylamide) (PNIPAM), hydrophobized polysaccharides, and hydrophobized pullulan, may self-assemble or aggregate to create nanogels in aqueous environments. Due to pullulan's facile chemical modification and non-toxic, non-immunogenic, non-mutagenic, and non-carcinogenic qualities, it is widely used in the food, cosmetic, and pharmaceutical sectors. Pullulan is non-immunogenic, blood-compatible, biologically compatible, and recyclable. It may also be chemically changed to have amphiphilic qualities. Pullulan's significant binding affinity to asialoglycoprotein receptors, which promotes receptor-mediated endocytosis, is another beneficial property. In particular, one group of researchers has looked at pullulan (CHP) nanogels that contain cholesterol [5].

These nanogels can combine with different proteins, medications, and DNA to create complexes. They may also be used to coat solid surfaces, including cells, liposomes, and particles. Additionally, hybrid nanogels can improve the way insulin and anticancer medications are delivered. CHP molecules self-aggregate to produce monodisperse stable nanogels by the interaction of hydrophobic groups, forming physical cross-linking sites. CHP is composed of a pullulan backbone with cholesterol branches [5].

Micellar nanogels: Micellar nanogels are created through the self-structuring of hydrophobic and water-soluble components or by utilizing branched copolymers in an aqueous solution. These nanogels typically include a core-crosslinking framework. The hydrophobic section envelops the hydrophilic polymer segment to create the core. The steady establishment of a core-shell structure around the tiny particles is facilitated by the hydrophilic segment, which is necessary for the creation of hydrogen bonds. This shell has excellent rigidity and is stimuli-responsive. Micellar nanogels provide a large surface area that may be used for drug accommodation through simple physical entrapment methods.

When succinylated poly(glycidol) is mixed with liposomes, they effectively deliver calcein into the cytoplasm through chain fusion at pH levels below liposomes, which resemble thermo- and pH-responsive nanogels like PNIPAAm, are under investigation for their potential in transdermal administration. Liposomes are small vesicles composed of a lipid bilayer structure surrounding an aqueous compartment, formed from lipid molecules. They consist of various components, with phospholipids and cholesterol being the primary constituents. Liposomes have been extensively employed as carriers for a wide array of substances, including anticancer agents, antibiotics, antifungals, antivirals, and bioactive macromolecules [6].

Liposome-modified nanogels: These nanogels serve as advanced drug carriers effective in treating a variety of diseases. They are physically cross-linked and responsive to stimuli, making them valuable for transdermal drug delivery applications due to their distinctive characteristics. The release of liposomes is influenced by factors such as pH and temperature. For example, liposomes incorporating succinylated poly(glycerol) efficiently deliver calcein to the cytoplasm at pH 5.5 [7].

Chemically cross-linked gels: Covalent bonds, which are permanent chemical bonds, are dispersed throughout the gel networks of chemical gels. These cross-linked gel systems' characteristics are determined by the particular chemical bonds and functional groups found in the gel networks. A variety of techniques, such as chain-growth polymerization, addition and condensation polymerization, and gamma and electron beam polymerization, can be used to create chemically cross-linked hydrogels. Processes including anionic and cationic polymerization, controlled free radical polymerization, and free radical polymerization that goes through initiation, propagation, and termination phases are all included in chain-growth polymerization. In the case of nanogel preparation, for instance, disulfide cross-linking is used, where pendant thiol groups facilitate environmentally friendly chemistry [8]. The choice of polymer for nanogel synthesis depends on the desired properties, such as biodegradability, stimuli-responsiveness, and targeted delivery, to meet the specific requirements of the biomedical application.

Methods of nanogel preparation

The choice of polymer for nanogel synthesis depends on the desired properties, such as biodegradability, stimuli-responsiveness, and targeted delivery, to meet the specific requirements of the biomedical application.

Emulsion Solvent Diffusion Method

An organic layer solubilizes the drug's aqueous solution. The drug phase is made by dissolving a polymer and a gelling agent in water, adding them dropwise to the aqueous phase, and homogenizing it for 30 minutes at 6000 rpm. When this combination is homogenized into nanodroplets using a homogenizer, an oil-water emulsion is produced. Triethanolamine is added to the oil-in-water emulsion and vigorously agitated at 8000 revolutions per minute for one hour to generate nanogel. Triethanolamine is added to the oil-in-water emulsion and vigorously agitated at 8000 revolutions per minute for one hour to generate nanogel [9].

Nanoprecipitated Method

The polymer precipitates out when the organic phase containing the medicine and polymer interacts with the surfactant aqueous layer. After the excess solvent is removed, polymeric nanoparticles remain. Once the particles are moistened, a gelling agent and the required amount of nanoparticle dispersion are added. Triethanolamine is used to stabilize the pH [9].

Evaporation of the Solvent Method

The drug-polymer mixture is injected into the specified area of the aqueous phase during the two-hour treatment, and a magnetic stirrer is used to continuously agitate the mixture at 1000 rpm. The drug-polymer mixture is injected into the specified area of the aqueous phase during the two-hour treatment, and a magnetic stirrer is used to continuously agitate the mixture at 1000 pm. The resulting nanosponges are then filtered and dried in a hot air oven at 40 °C for 24 hours. The nanosponges are placed into capsules for storage after they have dried. It is advised to submerge the polymer in water for two hours before initiating the gel-forming process to achieve a uniform dispersion. Following this, the polymer should be agitated at 6000 rpm. After utilizing a pH-modifying agent to correct the pH, the customized nanosponge suspension and permeation-enhancing agents are added to the aqueous dispersion [10].

Reverse Micellar Method

An organic solvent is used to dissolve a polymer, drug, or surfactant. After that, the agent that crosses links is introduced, and it is allowed to integrate overnight. After the nanoparticles are purified, the solvent is evaporated, resulting in a dried bulk. The gelling component is dissolved in water, and when the nanoparticles are combined with this aqueous phase containing the gelling agent, nanogel are formed. The pH is adjusted using a neutralizing substance [11].

Modified Diffusion Emulsification Method

The drug is blended precisely in a ratio with a polymer that has been coupled with the solvent. The organic phase is formed by constantly agitating the drug-polymer mixture at a rate of 5000 to 10,000 rpm in the aqueous phase. The organic component is added to the hydrostabilization solution at a rate of 0.5 mL per minute using a syringe fitted with a needle. The suspension is subjected to sonication for five to ten minutes following six minutes of stirring at 10,000 to 25,000 pm [12].

Loading techniques for drugs in nanogels

Loading drugs into nanogels is crucial for their application as effective carriers of therapeutic agents. Several methods are employed to achieve high-level drug uptake potential, thereby minimizing the carrier quantity Needed. These methods include the following:

Covalent Conjugation

Bioactive entities can be covalently conjugated to nanogels either during their synthesis or using preformed nanogels. For instance, acrylic groups can undergo modification through enzymatic interaction, followed by copolymerization with acrylamide in dilute aqueous solutions or inverse micro-emulsions to achieve nano-sized hydrogels [13].

Physical Entrapment

Pullulan nanogels treated with cholesterol allowed proteins to be physically trapped and integrated. Similarly, hydrophobic molecules were added to HA nanogels within non-polar domains to enclose SiRNA, creating a water-resistant chain in certain nanogels. For example, pullulan nanogels treated with cholesterol were able to solubilize pge E2. N-hexylcarbamoyl-5-fluorouracil (HCFU) was independently added to cross-linked nanogels made of N-vinylpyrrolidone (VP) and N-isopropylacrylamide (NIPAAm) copolymers (PNIPAAm/VP) in a different investigation. The method of physical entrapment relies largely on the nanocarriers' pore sizes and the enzyme molecules' sizes. Other approaches utilize active parts present on the outermost layers of nanocarriers and enzymes.

Self-Assembly

One example of a thermodynamically stable supramolecular structure with regular topologies and cognitive functions is biological self-assembly. This is a phenomenon involving the independent, spontaneous, and reversible organizing of molecular units into well-defined aggregates with energetically minimal flaws. Self-assembly has several benefits, including being adaptable, affordable, and occurring around thermodynamic minima, which produces sturdy, stable structures. An equilibrium of weak, non-covalent interactions, including hydrogen bonds, van der Waals, Coulombic, and hydrophobic forces, is necessary for the process to occur. Due to their multiple connections, molecules undergo diffusion followed by particular affiliations through non-covalent interactions, such as hydrophobic and electrostatic associations, which together control the meeting's structural and conformational behavior. Electrostatic attractions cause oppositely charged polysaccharides to join easily, whereas neutral polysaccharides interact less or not at all unless they are chemically changed to cause assembly. Hydrophobic interactions allow highly water-soluble polysaccharides to stimulate the production of nanoparticles. Three techniques can be used to create amphiphilic polymers: either use brick polymers with alternating hydrophilic and hydrophobic segments, or graft hydrophilic chains onto a hydrophobic backbone.

Amphiphilic polymers spontaneously establish intra- or intermolecular connections among hydrophobic moieties to create self-aggregated nanoparticles in aqueous settings, mainly to reduce interfacial free energy. Physically and chemically, these polymers align themselves to aggregate hydrophobic segments in the material's core while exposing hydrophilic portions to the aqueous media. The critical micelle concentration, also known as the critical aggregate concentration, is the level at which polymeric chains assemble [13].

Drug release mechanism of nanogels

Diffusion Mechanism

**Figure 4:**
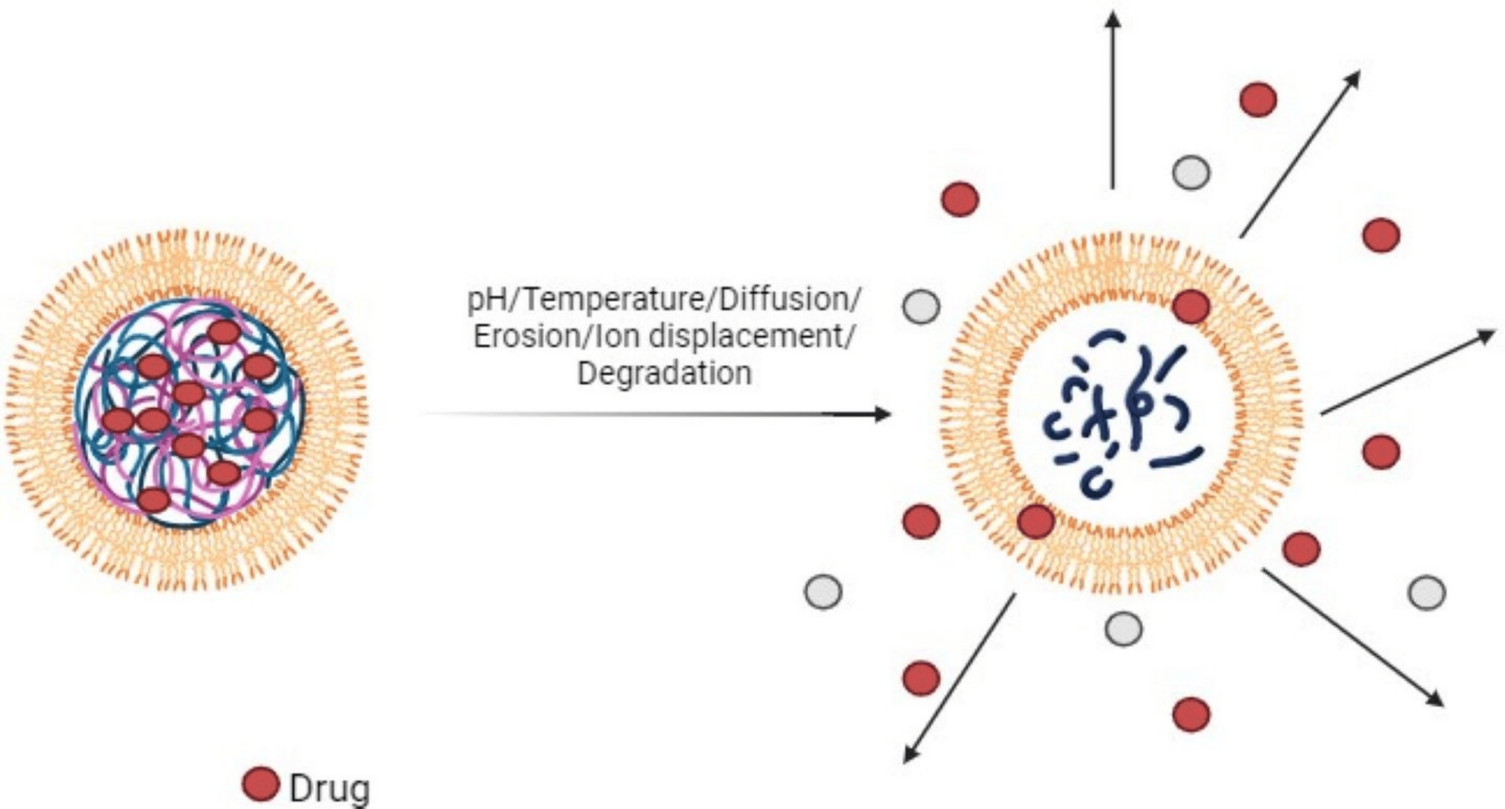
Drug release mechanism of nanogels This image is an original creation by author Ferozekhan S.

Doxorubicin release from persistent hydrogel nanoparticles composed of pluronic block copolymers has been shown to occur primarily through a diffusion-based mechanism. This simple release mechanism has been widely employed in the development of various nanomedicine formulations, including polymeric micelles, some of which have already progressed to clinical stages. The diffusion-controlled release from these hydrogel nanoparticles allows for the sustained and controlled delivery of the encapsulated doxorubicin, which is an important characteristic for enhancing the effectiveness of treatment and minimizing negative effects associated with this anticancer drug. Figure 4 shows the drug release mechanism of nanogels. The ability to modulate the release kinetics through the design of the nanoparticle composition and structure has made this diffusion-based approach a valuable strategy in the development of nanomedicine platforms for drug delivery.

Nanogel Degradation

Nanogels have been designed to exhibit pH sensitivity, which can trigger the release of encapsulated therapeutic agents in response to changes in the surrounding environment. For example, the number of diethylaminopropyl groups in glycol chitosan nanoparticles significantly increased. The excretion of the anticancer medication doxorubicin is due to the pH-responsive nature of these modifications. Similarly, cationic nanogels based on diethylaminoethyl methacrylate demonstrated significant alterations in mesh size in response to pH changes, facilitating the release of medium-sized molecules.

This pH-responsive behavior of nanogels allows for the gradual release of encapsulated drugs, such as doxorubicin, in a targeted manner. The degradation of these nanogels can be triggered by specific pH conditions, leading to the release of the therapeutic cargo. In addition, pH sensitivity can facilitate the elimination of vacant nanogel vehicles after the medication has been released, ensuring efficient clearance from the body. The ability to modulate the discharge kinetics of enclosed molecules, including drugs and fluorescent dyes, through pH-responsive degradation makes nanogels attractive candidates for precision drug delivery purposes. The enhanced release of doxorubicin from glycol chitosan nanoparticles and the mesh size alterations in diethyl aminoethyl methacrylate-based nano gels demonstrate the potential of this approach in controlling the release of therapeutics in response to specific environmental cues.

Displacement by Ions Present in the Environment

Nanogels have been engineered to respond to specific environmental stimuli, enabling the controlled release of their cargo at the desired target site. One example is the use of disulfide cross-linked poly (oligoethylene glycol methacrylate) (POEOMA) nanogels, which can undergo biodegradation into water-dissolvable polymers in the presence of glutathione, a tripeptide typically encountered within cells. This redox-responsive behavior allows for the selective release of the encapsulated therapeutic agents in the intracellular environment.

Another approach involves the use of cell membrane-triggered release mechanisms. Cationic nanogels can form complexes with negatively charged drugs, facilitating their cellular uptake. Once inside the cell, the drug can dissociate from the nanogel complex due to changes in the local environment, leading to enhanced accumulation of the therapeutic agent within the target cells. These stimuli-responsive nanogel systems offer a promising strategy for targeted drug delivery, as they can protect the encapsulated cargo during circulation and release it in a controlled manner upon reaching the desired site of action. The ability to design nano gels that respond to specific environmental cues, such as redox potential or pH changes, allows for the development of a more efficient and targeted drug delivery platform [14].

Characterization of nanogels

Dynamic Light Scattering (DLS) Analysis

One technique for figuring out the dimensions of the spread of nanoparticles in liquids is dynamic light scattering analysis. Light scattering is captured on the microsecond time scale during this operation. The assessment of the impact of cross-linkers and the prospective charge of polymer chains on nanogel size is aided by the effective hydrodynamic radius of particles. In addition, DLS can be used to examine how nanogels swell in different types of media. It is crucial to keep in mind that smaller polymer particles might not be completely accounted for in DLS data. Thus, it is frequently necessary to use a variety of analytical techniques to fully comprehend the features of nanogels.

Scanning Electron Microscopy (SEM)

Particle surfaces and sizes can be examined with electron microscopy. Nanogel morphological characteristics can be evaluated and particle sizes between fifty and eighty nm can be measured using SEM.

Circular Dichroism (CD) Spectroscopy

CD spectroscopy may determine the final product's optical activity. This method works particularly well for finding chiral compounds embedded in nanogels. These molecules produce spiral structures that give rise to macromolecular formations that CD can detect. These structures have a chiral core.

Size-Exclusion Chromatography

Size-based exclusion materials are separated using the chromatography (SEC) process according to their size. Its main purpose is to ascertain the molar mass spread of the nanogel and the molar volume of its fractions.

Field-Flow Fractionation (FFF)

The separation method known as field-flow fractionation (FFF) applies a cross-flow to a suspension or solution that is pumped via a long, narrow channel. The primary flow direction and this cross-flow direction are perpendicular. The ability of FFF to separate polymer compounds with excellent resolution over a wide colloidal size range sets it apart from other separation techniques. The method is based on the longitudinal movement of particles in the solution, in which the components move at different rates according to their mass or size, causing separation as a result of these differing speeds.

Nanoparticle Tracking Analysis

The technique known as nanoparticle tracking analytics (NTA) is used to size particles with a diameter of between 30 and thousand nm. It makes it possible to see and record nanoparticles in a solution by fusing charge-coupled device (CCD) microscopy with laser light scattering microscopy. When a single nanoparticle moves in Brownian motion, NTA can detect it, track it, and determine how big it is. This method quantifies the concentration and size of the particles [15].

Swelling Studies of Nanogels

One important characteristic of nanogels is expansion, which is characterized by their capacity to absorb aqueous solutions or water. Weighing the nanogels is the easiest way to evaluate the kinetics and equilibrium of swelling; the weight of the swelled nanogel relative to its original weight may be used to measure the degree of swelling. The type and structure of the monomer, connect volume, pH, temperatures, and ionic strength are some of the factors that affect nanogel swelling [15].

Applications of nanogel

Anti-Inflammatory Action

The bilayered nanoparticles are composed of poly(lactide-co-glycolide) and chitosan, with an oleic acid-coated surface. Following that, these nanoparticles were added to nanogels that were made with carbopol and hydroxypropyl methylcellulose (HPMC). Two anti-inflammatory medications, spantide II and ketoprofen, were administered topically using the nanogel technology to treat inflammatory skin conditions such as psoriatic plaque and allergic contact dermatitis. The results showed that the nanogel formulation enhanced the potential for percutaneous delivery of anti-inflammatory drugs to the deeper skin layers, thereby improving their effectiveness in treating various skin inflammatory conditions [7].

Diabetes Treatment

Injectable nanogel systems have shown promising applications in the treatment of diabetes by providing a self-regulated insulin delivery mechanism [5]. These nanogels are designed to be sensitive to changes in blood glucose levels and have the ability to secrete insulin in response to hyperglycemic conditions. The nanogel system typically consists of oppositely electrostatically charged nanoparticles that attract each other, forming a mechanically strong gel matrix. Dextran spheres packed with glucose-converting enzymes, such as glucose oxidase (GOx) and catalase (CAT), are incorporated into the nanogel. When blood glucose levels are elevated, glucose diffuses through the gel matrix, and the enzymes convert dextrose into gluconic acid, lowering the pH of the surrounding environment. The pH change triggers the dissociation of the dextran spheres, leading to the release of insulin from the nanogel system.

Preclinical animal studies have demonstrated the efficacy of these glucose-responsive nanogels in regulating blood glucose levels in diabetic animal models. For instance, a single subcutaneous injection of the nanogel system in type 1 diabetic mice has been shown to maintain normal blood glucose levels for up to 10 days. However, further research is still needed to optimize the delivery properties and dosage requirements for potential human trials.

Uses in Cancer Therapy

A lot of research has been done on nanogels as drug-delivery vehicles for cancer treatment. They have been loaded with a variety of anticancer medications, including temozolomide, cisplatin, 5-fluorouracil, heparin, and doxorubicin, to target malignancies of the liver, breast, prostate, and lung. The goals of using nanogels in cancer treatment are to increase therapeutic efficacy, decrease damage to nearby healthy tissues, and accomplish targeted drug delivery. For example, the primary application of cisplatin-loaded thermo- and pH-responsive nanogels in the treatment of breast cancer has been studied. Reducible nanogels containing heparin have been utilized to induce apoptosis in melanoma cells. Furthermore, it has been demonstrated that fludarabine-encapsulated polyplex nanogels enhance the activity and decrease the cytotoxicity of fludarabine [10].

Nanogels in Vaginal Drug Delivery

Applications for nanogels in vaginal medication delivery have shown promise. Various vaginal infections have been treated with antibacterial medications included in vaginal nanogels. In addition to relieving other sexual difficulties, these nanogel formulations can help lessen vaginal discomfort and discharges. It's crucial to remember, nevertheless, that using vaginal nanogels throughout menstruation and pregnancy is not advised due to a few disadvantages. To lower the risk of HIV infection in women, researchers have also looked into the usage of nanogels containing antiretroviral medications [16]. Using a two-step desolvation process, for example, the hydroxypropyl methylcellulose compound (HPMC K15M) has been formed into gelatin nanoparticles for tenofovir, a vaginal gel being explored for HIV prophylaxis, while also acting as a bioadhesive polymer and gelling agent [16]. The resulting nanogel formulation demonstrated enhanced bio adhesion and improved membrane permeability, suggesting its potential for effective vaginal drug delivery [16].

Nanogels in Ophthalmic Drug Delivery

In the field of ophthalmic drug delivery, nanogels have been explored as a promising platform. created a pH-sensitive polyvinyl pyrrolidone-poly acrylic acid nanogel by polymerizing acrylic acid in an aqueous solution of polyvinyl pyrrolidone by γ-radiation-induced polymerization. The purpose of this nanogel system was to encapsulate the drug pilocarpine, which is used to treat various eye conditions, to maintain satisfactory Extended retention of the drug at the target site [17]. The use of a pH-sensitive nanogel system allows for the controlled release of pilocarpine, ensuring that a sufficient amount of the concentration of the drug is maintained at the target site in the eye for a prolonged duration. This approach can improve therapeutic efficacy and patient compliance by reducing the frequency of administration required compared to conventional eye drop formulations.

Nanogels in Bleeding Blockage

Nanogels for stopping bleeding blood clotting have been achieved even in dire circumstances by using a protein-loaded nanogel. The proteins combine to form a biodegradable gel on the nanocarrier [18].

Nanogels in Neurodegenerative Diseases

Nanogels have become a viable method for administering drugs for the treatment of neurodegenerative diseases. These nanoscale hydrogel particles, composed of cross-linked polyethylene glycol and polyethyleneimine, can effectively encapsulate and deliver negatively charged oligonucleotides (ODNs) to the brain [19]. The formation of a stable polyelectrolyte complex between the nanogel and the ODN, with particle sizes less than 100 nm, facilitates their passage through the blood-brain barrier (BBB). Furthermore, the surface modification of these nanogels with targeting ligands, such as transferrin or insulin, has been shown to enhance their transport efficiency across the BBB [19].

Vaccine Delivery Using Nanogels

One of the most important methods for eliciting immune responses specific to antigens is vaccination. Polymeric nanogels have become an innovative and alternative vaccine delivery method to improve the efficacy and performance of vaccinations. The capacity of nanogels to shield vaccine antigens from enzyme breakdown within the nanogel network is one of the vaccine delivery system's benefits. Furthermore, by attaching antibodies and other specific ligands to the surface of the nanogels, the target specificity of vaccine administration can be greatly increased. This enhances the vaccination's overall effectiveness by enabling the vaccine antigens to be delivered selectively to the targeted immune cells or tissues. Compared to conventional vaccine formulations, the use of nano gels as a delivery system offers the potential to enhance the stability, targeting, and controlled release of the vaccine components, ultimately leading to more effective and potent immune responses [6].

Transdermal Drug Delivery

Compared to alternative administration methods, transdermal drug delivery has several benefits, including avoiding first-pass metabolism, increasing patient compliance, preserving constant state dosage levels in plasma, and boosting medication efficacy. Many strategies have been investigated to improve the absorption of drugs into the target region, such as topical administration of active medicinal components to the corneum region via nanogels.

Aceclofenac, a non-steroidal anti-inflammatory drug, is commonly administered orally, but this route can cause side effects like ulcers and gastric bleeding. Transdermal aceclofenac delivery has been studied as a potential workaround for this problem. An aceclofenac emulsion was created and added to a gel matrix using the emulsion-based solvent diffusion method to create a nanogel preparation for transdermal drug administration. This nanogel system demonstrated improved stability and permeability compared to conventional formulations, making it a promising strategy for the transdermal administration of aceclofenac while minimizing gastrointestinal side effects associated with oral administration [20].

Future perspectives

Nanogels hold great promise as advanced drug delivery systems due to their ability to shield payloads from degradation and release drugs in a controlled manner. They are being explored for various therapeutic applications, including treating central nervous system disorders, cancer, diabetes, and inflammatory diseases, owing to their capacity to cross the BBB and target specific sites. The customization of nanogels to respond to environmental stimuli enhances their precision and efficiency. Continued advancements in their synthesis and characterization are expected to improve their efficacy and stability. As research progresses, the transition to clinical trials and ensuring the scalability and cost-effectiveness of nanogel production will be crucial for their commercial viability. Beyond drug delivery, nanogels have potential applications in ocular conditions, nasal and vaginal drug delivery, water decontamination, and catalysis, showcasing their versatility and broad scope. Overall, nanogels are positioned as key players in the next generation of drug delivery systems and beyond, with ongoing research paving the way for their expanded use in various fields.

## Conclusions

Nanogels represent a versatile and promising approach to drug delivery, offering unique advantages such as adjustable dimensions, simplicity of production, compatibility with biological systems, and reactivity to environmental stimuli. Their ability to encapsulate multiple bioactive ingredients in the same carrier makes them ideal for various drug delivery applications. Nanogels can protect the encapsulated cargo during circulation and release it in a regulated way upon reaching the desired site of action, thanks to their stimuli-responsive behavior. Overall, the comprehensive review demonstrates the significant potential of nanogel-based drug delivery systems to revolutionize the field of therapeutics.
